# Outcomes of three-piece rigid scleral fixated intraocular lens implantation in subjects with deficient posterior capsule following complications in manual small incision cataract surgery

**DOI:** 10.1016/j.heliyon.2023.e20345

**Published:** 2023-09-20

**Authors:** G Nageswar Rao, Sonu Kumar, Nidhi Sinha, Bhumika Rath, Arttatrana Pal

**Affiliations:** a Department of Ophthalmology, Kalinga Institute of Medical Science, Kalinga Institute of Industrial Technology, Bhubaneswar, Odisha 751024, India; b Vision Care, Center for Retina, Bhubaneswar, Odisha 751024, India; c Department of Zoology, School of Life Sciences, Mahatma Gandhi Central University, Motihari, Bihar 845401, India

**Keywords:** Intraocular lens, Pars plana vitrectomy, Scleral fixation, Cataract

## Abstract

**Objective:**

To evaluate the surgical visual outcomes of three-piece rigid scleral fixated intraocular lens (SFIOL) implantation in subjects with deficient posterior capsule following complications of cataract extraction.

**Design:**

Retrospective 4-year cohort study.

**Participants:**

Data from 174 eyes that underwent SFIOL combined with pars plana vitrectomy (PPV) between January 2018 and March 2022 and follow-up exams were included.

**Methods:**

Demographic characteristics including primary indications for surgery, history of trauma, laterality, baseline and best-corrected visual acuity (BCVA), refraction as spherical equivalent (SE), intraocular pressure (IOP), duration of follow-up, and complications were analyzed.

**Results:**

The mean preoperative BCVA was 1.38 ± 0.46 logarithm of the minimum angle of resolution (logMAR), which improved significantly to 0.37 ± 0.22 logMAR. The baseline refractive status measured in spherical equivalent (SE) was 4.1 ± 6.2 Diopters (D), and the postoperative status was −0.4 ± 0.97 D. Early postoperative complications included hypotony (n = 1; 0.57%, vitreous hemorrhage (n = 3; 1.72%), elevated IOP (n = 8; 4.59%), mild dilated pupil (n = 1; 0.57%) and corneal edema (n = 16; 9.19%). Late complications included in this study were retinal detachment (n = 1; 0.57%), cystoid macular edema (CME) (n = 1; 0.57%), primary glaucoma (n = 1; 0.57%), secondary glaucoma (n = 13; 7.47%), zonular dehiscence (n = 3; 1.72%), retinal pigment epithelium (RPE) changes (n = 3; 1.72%), choroidal coloboma (n = 2; 1.14%), posterior dislocation of posterior chamber IOL (PCIOL) (n = 1; 0.57%), corneal decompensation (n = 1; 0.57%), retinal hemorrhage (n = 1; 0.57%), macular hole (n = 1; 0.57%), chronic uveitis (n = 1; 0.57%), mild non-proliferative diabetic retinopathy (NPDR) (n = 3; 1.72%), and mild NPDR with diabetic macular edema (DME) (n = 1; 0.57%).

**Conclusion:**

Integrating IOL implantation with vitrectomy various posterior segment complications were resolved in the same setting without attempting a second surgery.

## 1 Introduction

One of the most widely used and effective potential surgeries among subjects with an intraocular lens (IOL) implant is cataract surgery [1]. Even expert surgeons may find it extremely difficult to perform IOL when capsular support is poor or lacking. Recently, multiple approaches have been used to cope with zonular dehiscence, and most of the techniques used for iris-claw or iris fixation suturing [[2], [3], [4], [5], [6]], anterior chamber IOL (ACIOL) implantation [7], scleral fixation of the intraocular lens (SFIOL) with suturing [8], sutureless intrascleral fixation of the IOL, and glued IOL [2,[9], [10], [11]]. However, haptic slippage and IOL dislocation are common challenges for surgeons. An earlier study demonstrated sutureless intrascleral fixation [9] accompanied by Gabor Scharioth's tunnel fixation methodology, which was later improved to glued transscleral fixation [10,11]. Additionally, studies have shown that the SFIOL implant is perceived as a reliable surgical procedure and reduces suture-related comorbidities [12]. Moreover, studies have reported that closed-globe injury consequences have previously been treated with a combination of pars plana vitrectomy (PPV) and scleral fixation of a rigid IOL. However, it is a frequent complication associated with eyesight issues [13].

Lens-related issues are prominent reasons for vitreoretinal therapy. However, the clinical outcomes of combined SFIOL and PPV 4-point suture fixation of a foldable IOL are safe and visually satisfactory procedures [14]. However, during the surgical procedure, transscleral fixation using the suture approach in the ciliary sulcus and pars plana is a critical technique. Many clinical studies have shown in long-term investigations that there are some serious postoperative concerns, including transient hypotony, suture exposure and degradation, and endophthalmitis [[15], [16], [17], [18], [19]]. Many subjects were initially concerned about an increased risk of diabetic retinopathy (DR) progression after cataract extraction because the lens was assumed to operate as a protective wall to protect anterior compartment neovascularization [[20], [21], [22]]. Different alternatives for exteriorizing and securing haptics into scleral pockets have been explored [23]. However, in all the strategies mentioned for haptic stabilization, carry the concern of unplanned haptic rebounds into the cavity of the vitreous and intraoperative IOL drops. The benefits and drawbacks of each technique have been described in many previous and recent studies. Insight restoration of aphakic eyes, SFIOL is essential because of the lack of capsular support.

Recently, ACIOL, iris-claw IOL, and sutured and sutureless SFIOL approaches have become surgical options for aphakia correction. The Yamane technique-based SFIOL is a unique, relatively efficient, safe, and simple sutureless SFIOL approach [24]. Moreover, this procedure was performed with a secondary corrective surgery for aphakic eye restoration. One report revealed intermediate outcomes using the SFIOL's modified Yamane methodology [25]. During the surgical procedure, any other physiological complications could be the reason behind the anterior and posterior segment issues in ocular injuries. Traumatic cataracts and subluxated and even dislocated IOLs are examples of anterior segment problems [[26], [27], [28]]. These complications can lead to corneal injuries, vitreous hemorrhage, traumatic ocular neuropathy, choroidal rupture, and other consequences. The proper handling of these issues is generally challenging, and visual outcomes are unsatisfactory [29]. Due to the lack of appropriate zonular and capsular integrity, posterior chamber of intra-ocular lens (PCIOL) insertion after cataract and removal of the lens are generally challenging in severe traumatic situations. Additional choices for traumatic aphakia include ACIOL, iris-fixated IOL, and SFIOL. Moreover, ACIOL and iris-fixated IOL were associated with a higher risk of cystoid macular edema (CME), secondary glaucoma, postoperative uveitis, peripheral anterior synechiae, corneal endothelial decompensation, and other comorbidities. With the orientation of the IOLs within a more posterior plane, the implantation of the SFIOL helps to address these disadvantages.

Sutured and sutureless SFIOLs were used in SFIOL insertion procedures. Suture breakage, exposure to suture knots, with other postoperative complications may be related to suture SFIOL [30].To minimize such knot-related complications, further many other techniques carried out recently for better surgical outcomes [[31], [32], [33], [34]]. As vitreoretinal and cataract operating innovations and improved technologies arise, more medical evidence is crucial to analyze the updated surgical success of SFIOL in vitrectomized eyes. PPV combined with SFIOL appears to be a promising therapeutic option for these subjects. However, the effectiveness and safety of this combination surgical approach remain debatable. This study aimed to investigate the surgical consequences of PPV with the SFIOL technique, as well as the complications that arise during and after surgery.

## 2 Methods

### 2.1 Study population

This retrospective, non-comparative case study of 174 subjects was conducted at the Vision Care Center for Retina, Bhubaneswar, India. A retrospective chart review was performed for all subjects who underwent combined PPV and SFIOL surgery between January 2018 and March 2022 with a follow-up at least six months. The institutional review board of Kar Vision Eye Hospital, Bhubaneswar, India examined and approved the study protocol (ECR/1630/Inst/OD/2021-B), which adhered to the guidelines of the Declaration of Helsinki.

### 2.2 Inclusion criteria

Subjects with open globe injury (OGI) and closed globe injury (CGI) attributable to the posterior lens or IOL dislocation who have had traumatic aphakia. The types of ocular injuries, such as OGI or CGI, in addition to data about previous ocular surgeries, such as globe repair, prior IOL surgery, prior retinal detachment (RD) surgery, and so forth, were among the investigated parameters. The existence of ophthalmologic comorbid conditions such as vitreous hemorrhage, choroidal rupture, glaucoma, traumatic optic neuropathy, corneal or scleral injury, intraoperative, and postoperative complications of consolidated PPV with SFIOL. Subjects who fulfilled the inclusion criteria and provided written informed consent were obtained before any *ophthalmic* procedure. The study examined demographic characteristics including primary surgical indications, past trauma history, laterality, concomitant systemic syndromes, associated posterior segment diseases, pre-and postoperative best-corrected visual acuity (BCVA), slit lamp examination, type and power of the lens, pre-and postoperative intraocular pressure (IOP) assisted with applanation tonometry and indirect ophthalmoscopy, postoperative follow-up time, refraction, IOL orientation, and co-existence of any problems was among the postoperative information recorded at 6 months and the last visit.

### 2.3 Surgical technique

Scleral fixation of the IOL with PPV was planned because of the non-availability and inadequacy of capsular support on slit-lamp examination. During the SFIOL implantation period, all subjects underwent PPV. For every eye, the mean number of operations was 1. All ophthalmological interventions were performed by the same surgeon using the criteria based on a machine, the Constellation ®Vision System (Alcon, Fort Worth, Texas, USA), assisted with 25 G instrumentation.

#### 2.3.1 Scleral pockets

The patient underwent peribulbar anesthesia with 5 mL each of lignocaine hydrochloride (2%) and bupivacaine hydrochloride (0.5%), and the surgical operation was carried out most efficiently and effectively feasible. Further, the sclera-specific area was identified with the application of gentian violet stain at 1.5 mm and 3.0 mm mostly from the limbus just after selective peritomy, which was accomplished at 3 o'clock and 9 o'clock positions respectively, and two parallel, partial-thickness radially having scleral incisions were made in between these two markers. This partial thickness was made of the scleral flap and raised by performing lamellar dissection between two incisions using a crescent-shaped blade. Subsequently, the bleeding vessels were cauterized and 1 mm tunnels were created perpendicular to the tiny corner of the rectangle in the center, assisted with a 26 G needle across both sides, with the orientation of the tunnel aligned at the desired location of the IOL haptics.

The vitrectomy-designed ports were directed away from the scleral flaps in addition to the standard 25 G PPV and the infusion cannula was positioned in the inferotemporal region. 25 G PPV was done in all cases. In cases with posteriorly dislocated IOL, the IOL was removed through the limbal incision. A fresh three-piece IOLs was used for scleral fixation adopting the Agarwal technique [35] but without using the glue. For additional strengthening of haptic at the scleral groove used a 10-O nylon suture. In other cases, the dropped IOL was repositioned. Phaco-fragmentation of the dropped lens was performed using linear ultrasound power. In all subjects, the perfluorocarbon liquid was intended to safeguard the posterior pole and facilitate surgical intervention. Therefore, the IOL was implanted once the ports had been plugged.

#### 2.3.2 SFIOL

**Fig. 1:**
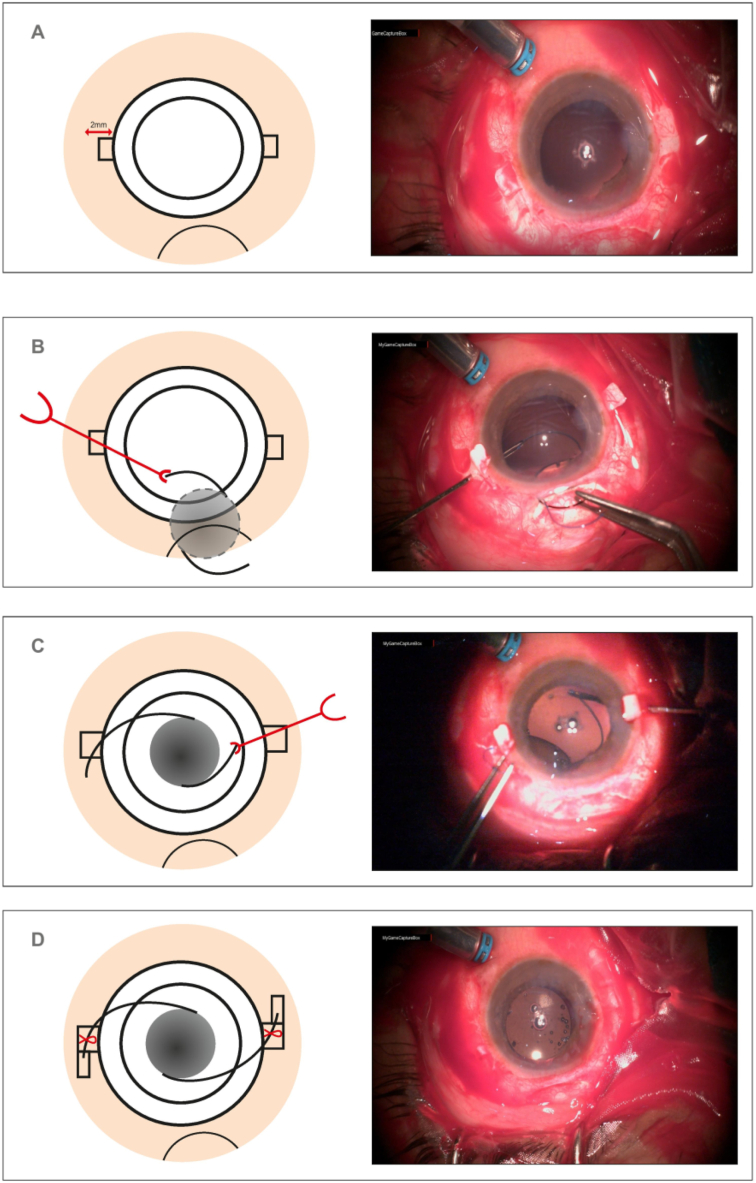
Key surgical steps: A – Partial thickness limbal based scleral flaps (0 and 180°). B – Leading haptic tip held with microforceps. C – Trailing haptic tip held with microforceps. D - Tip of haptics tucked in scleral tunnel and suture put.

Two sclerotomies were created in the flap beds using a 23 G micro-vitreoretinal blade (Alcon, Fort Worth, Texas, USA). Moreover, 1.5 mm as from the limbus region, and using a keratome, a 3.2 mm cut was executed at 12 o'clock. Subsequently, a three-piece foldable IOL (AcrySof MA60AC, Alcon Laboratories) was placed in the anterior chamber, along with an IOL shooter, to keep the trailing haptic from entering the anterior chamber. Further, the leading haptic of the IOL was directed into the serrated tip with a 25 G intravitreal forceps end-grip and externalized further via left sclerotomy. The trailing haptic was subsequently forced within the eye using McPherson forceps and externalized into the right sclerotomy. The haptic tips were then inserted into the scleral tunnel, which had been dissected. Sutures were then used to fix scleral flaps. Sutures were used to fix the vitrectomy port sites if they were leaking; otherwise, they were left sutureless. Furthermore, sutures were used to fix the conjunctival flaps. The key surgical steps are shown in Fig. 1.

#### 2.3.3 IOL centration

**Fig. 2:**
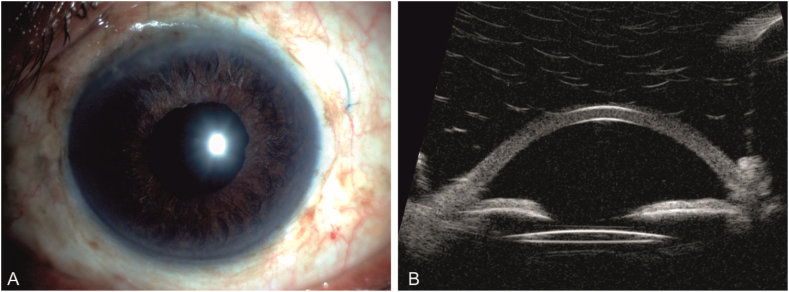
Representative figures showing the postoperative stability of SFIOL at the end of 6 months. (A) Slit lamp image. (B) Ultrasound biomicroscopy (UBM) image.

Proper alignment of the IOL center was verified, and Purkinje images of the cornea and IOL were captured intraoperatively using coaxial illumination from a microscope following eye repressurization. Further confirmation of the proper alignment of the first primary and third Purkinje pictures was performed by employing images recorded from the anterior surfaces of the cornea and IOL, respectively. This ensured that the existing IOL was precisely centered [Fig. 2], and it is a superior strategy to rely on an assessment of the cornea or pupil's center, which can be distorted far away from its actual visual axis. The pupil was entirely dilated after surgery, and the separation between the posterior surface of the iris and the nasal and temporal edges of the optic was calculated to check and confirm the tilt of the IOL. In addition, the entirely dilated pupil, the length between the nasal and temporal borders of the optic, and their respective limbus were measured in order to distinguish between these two, allowing a reliable assessment of the degree of orientation of IOL decentration.

2.4. Subjects follow up.

Each patient underwent at least 6 months of follow-up and was evaluated after surgery on days 1, 7, and 30 and monthly thereafter. Pre- and postoperative BCVA, IOP, IOL centration and retinal conditions were monitored at each follow-up. During the analysis process, special attention was paid to visual outcomes, as well as any issues that occurred during or after the procedure. At every visit, spectral-domain optical coherence tomography (Cirrus; Zeiss Meditec, Inc., Dublin, CA, USA) was performed to monitor macular edema.

### 2.4 Statistical analysis

IBM SPSS software (version 26.0; IBM Corp., Armonk, NY, USA) was used for the statistical analysis. The pre- and postoperative BCVA in logMAR and IOP in mmHg units were assessed using the application of a paired *t*-test. The variables' means and standard deviations were denoted as mean ± SD, and a p-value of ≤0.05 was taken to determine statistically significant.

## 3 Results

### 3.1 Demographic characteristics

Our study included 174 eyes of 174 subjects who had undergone SFIOL combined with PPV surgery (Table 1). The mean postoperatively follow-up time was 14.2 months (range = 6–50 months). The mean presenting age was 58.97 ± 17.57 years (range = 10–90 years), and 109 subjects were male (62.6%) and 65 female (37.4%). Out of the 109 subjects, the number of left eyes was 47.7% (N = 83), and the right eyes was 52.3% (N = 91). The common primary indication among the subjects undergoing surgery was aphakia, followed by posterior dislocation of the *PCIOL*, posterior dislocation of the IOL, PCIOL with macular hole, posterior dislocation of the IOL with vitreous hemorrhage, subluxation, *PCIOL*, traumatic, secondary glaucoma, nucleus drop, phacodomesis, hyphema, dialysis aphakia with vitreous hemorrhage, and other associated ocular indications (Table 1). The associated indications included the following: postoperative posterior capsular rent (PCR), PCR with hyphema, traumatic lens dislocation of lens matter, dislocation of cataract lens, retained lens matter with small pupil, SFIOL displacement, iris coboloma with subluxated cataract, PCIOL vitreous AC with corneal Descemet folds, posterior dislocation of lens matter, dislocation of lens fragments, and grade 5 nuclear sclerosis.

**Table 1:** Demographic and ocular features of preoperative indications for surgery posterior chamber intraocular lens (PCIOL) and intraocular lens (IOL).

Demographic Parameters	Characteristics	Values
Number of eyes (n)		174
Gender	Male	109 (62.6%)
	Female	65 (37.4%)
Age	Mean	58.97 ± 17.57 years
Follow up	Mean	14.2 (range = 6–50) months
Indications		
APHAKIA		48 (27.58%)
POSTERIOR DISLOCATION	POSTERIOR DISLOCATION OF PCIOL	18 (10.34%)
	PCIOL + MACULAR HOLE	1 (0.57%)
	POSTERIOR DISLOCATION OF IOL	17 (9.77%)
	IOL + VITROUS HEMORHAGE	1 (0.57%)
SECONDARY GLAUCOMA		6 (3.44%)
SUBLUXATION PCIOL		9 (5.17%)
TRAUMATIC		16 (9.19%)
PHACODOMESIS		
	HYPERMATURE CATARACT	2 (1.14%)
	TRAUMATIC CATARACT	3 (1.72%)
NUCLEUS DROP		
	TRAUMATIC	1 (0.56%)
	SURGICAL	5 (2.87%)
HYPHEMA		
	HYPHEMA + SECONDARY GLAUCOMA	1 (0.56%)
	HYPHEMA + VITROUS HEMORRHAGE	1 (0.56%)
IRIDODIALYSIS APHAKIA (VITREOUS HEMORRHAGE)		1 (0.56%)
OTHER ASSOCIATED OCULAR COMORBIDITIES		44 (25.28%)

### 3.2 Surgical outcomes

**Fig. 3:**
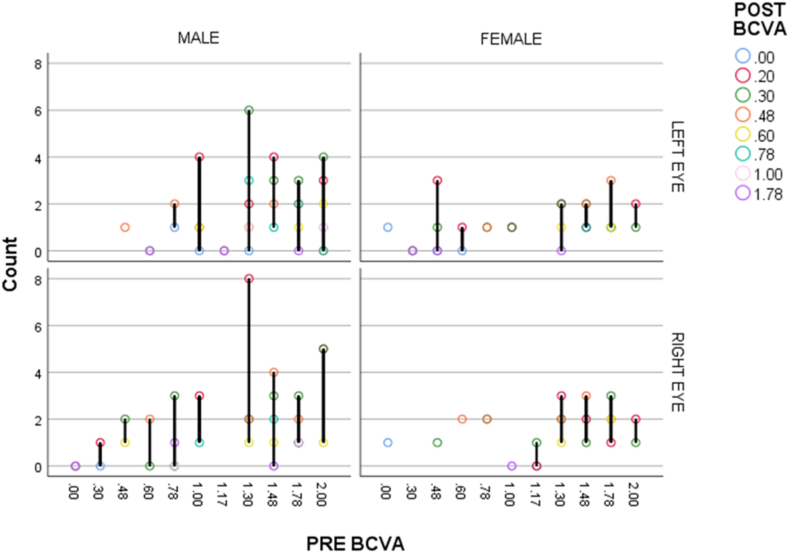
Details of subjects demographics and ocular features along with best corrected visual acuity (BCVA) preoperatively and at the last follow-up visit.

The mean preoperatively BCVA was 1.38 ± 0.46 logMAR units, and the mean postoperatively BCVA improved significantly at 3 months to 0.37 ± 0.22 logMAR units (p < 0.0001, t = 2.226) (Table 2). Our study showed that the number of eyes that had preoperative BCVA less or equal to 0.3 or better was 3 (1.7%), which was improved the total number of eyes to 110 (63.2%) postoperatively. The value of the BCVA range (0.4–1) was found in 39 (22.4%) eyes preoperatively, which was raised in the total number of eyes in 62 (36.2%) postoperatively. The value of BCVA worse than 1 before surgery in 132 (75.9%) eyes, which dramatically reduced the total number to 1 (0.6%) postoperatively after follow-up [Fig. 3]. Mean preoperatively and postoperatively IOP was 17.63 ± 6.66 mmHg and 15.72 ± 3.52 mmHg respectively at the 6-week follow-up (p < 0.0001, t = 3.826) (Table 3). The value of preoperatively IOP ranges of 5–15, 15–30, 30–40, 40–53 were observed in 72 (41.4%), 93 (53.4%), 5 (2.9%), 4 (2.3%) eyes, respectively, and the value of postoperatively IOP ranges of 7–15, 15–20, 20–30, 30–38 were observed in 76 (43.7%), 89 (51.1%), 7 (4.1%), 2 (1.10%) eyes, respectively. The baseline subject's refractive status was measured in terms of spherical equivalent (SE) and that was 4.1 ± 6.2 Diopters (D), and postoperatively the existing refractive status was −0.4 ± 0.97 D among the subjects.

**Table 2:** Statistical and clinical description of visual acuity preoperatively and at last follow-up visit.

	Preoperative		At last follow -up	Statistics
BCVA (logMAR)	Mean	1.38 ± 0.46	0.37 ± 0.22	p < 0.0001, t = 2.226
	0.3 or better	3 (1.7%)	110 (63.2%)	
	0.4–1	39 (22.4%)	62 (35.6%)	
	Worse than 1	132 (75.9%)	2 (1.2%)	
	Total	174 (100%)	174 (100%)	

**N.B:** BCVA, best-corrected visual acuity; logMAR, Logarithm of The Minimum Angle of Resolution.

**Table 3:** Statistics and clinical descriptive results of IOP preoperatively and at last follow-up visit.

	Preoperative		Last follow- up		Statistics
IOP (mm of Hg)	Mean	17.63 ± 6.66	15.72 ± 3.52		p < 0.0001, t = 3.826
	5–15	72 (41.4%)	7–15	76 (43.7%)	
	15–30	93 (53.4%)	15–20	89 (51.1.%)	
	30–40	5 (2.9%)	20–30	7 (4.1%)	
	40–53	4 (2.3%)	30–38	2 (1.1%)	
	Total	174 (100%)		174 (100%)	

N.B.: IOP: Intraocular pressure.

### 3.3 Surgical complications

Early postoperative complications included the subjects with hypotony as (n = 1; 0.57%), vitreous hemorrhage (n = 3; 1.72%), transiently raised IOP (n = 8; 4.59%), mildly dilated pupil (n = 1; 0.57%), and corneal edema (n = 16; 9.19%) (Table 4). Moreover, the late complications included retinal detachment (n = 1; 0.57%), CME (n = 1; 0.57%), sphincter tear (n = 6; 3.44%), primary glaucoma (n = 1; 0.57%), secondary glaucoma (n = 13; 7.47%), zonular dehiscence (n = 3; 1.72%), retinal pigment epithelium (RPE) changes (n = 3; 1.72%), iris coloboma (n = 2; 1.14%), choroidal coloboma (n = 2; 1.14%), posterior dislocation of PCIOL (n = 1; 0.57%), posterior synechiae (n = 2; 1.14%), corneal decompensation (n = 1; 0.57%), retinal hemorrhage (n = 1; 0.57%), macular hole (n = 1; 0.57%), chronic uveitis (n = 1; 0.57%), mild non-proliferative diabetic retinopathy (NPDR) (n = 3; 1.72%) and mild NPDR with diabetic macular edema (n = 1; 0.57%) (Table 4). Along with these complications, a case of posterior capsule opacification (n = 1; 0.57%), postoperative corneal scarring or opacity (n = 1; 0.57%), and marphanoid features (n = 1; 0.57%) were also observed (Table 4). However, no intraoperative complications were observed in our current study. Furthermore, observation among subjects with vitreous hemorrhage, employing topical and oral IOP-lowering agents for elevated IOP, as well as hypertonic saline drops along with topical steroids in case of ocular edema were all used to treat various ailments. Topical nonsteroidal anti-inflammatory agents were used to manage CME, and IOL refixation was performed in two eyes (1.14%) involving IOL decentration and tilting.

**Table 4:** Early and late postoperative complications.

Parameters	Values (n %)
POSTOPERATIVE COMPLICATIONS	
**CORNEAL EDEMA**	16 (9.19%)
**ZONULAR DEHISCENCE**	3 (1.72%)
**GLAUCOMA**	
*PRIMARY GLAUCOMA*	1 (0.57%)
*SECONDARY GLAUCOMA*	13 (7.47%)
**SPHINCTER TEAR**	6 (3.44%)
**BLUNT TRAUMA + VITROUS HEMORRHAGE**	1 (0.57%)
**RETINAL TEAR**	
*POSTERIOR DISLOCATION PCIOL*	1 (0.57%)
*CHOROIDAL COLOBOMA*	2 (1.14%)
*RPE CHANGES*	3 (1.72%)
*IRIS COLOBOMA*	2 (1.14%)
*MILD NPDR*	3 (1.72%)
*CHRONIC UVEITIS*	1 (0.57%)
*MACULAR HOLE*	1 (0.57%)
*MILD NPDR + DME*	1 (0.57%)
*CYSTOID MACULAR EDEMA*	1 (0.57%)
*RETINAL DETACHMENT*	1 (0.57%)
*VITREOUS HEMORRHAGE*	2 (1.14%)
*RETINAL HEMORRHAGE*	1 (0.57%)
*CORNEAL DECOMPENSATION*	1 (0.57%)
*POSTERIOR SYHECHIAE*	2 (1.14%)

**N.B.:** RPE: Retinal pigment epithelium; NPDR: Non-proliferative diabetic retinopathy; DME: Diabetic macular edema.

### 3.4 Techniques: PPV and SFIOL

Based on the inadequacies of existing capsular support for slit-lamp monitoring, SFIOL combined with PPV was designed. Descriptions of all the techniques among the recruited subjects are depicted in (Table 5). Each patient had a positive predictive value at the onset of SFIOL implantation. The mean number of surgeries performed was 1. The majority of subjects eyes underwent PPV + SFIOL [flap] (n = 100; 57.47%) and PPV + SFIOL [flapless] (n = 10; 5.74%). Along with PPV and SFIOL techniques surgeries undergoes additional IOL explanation with suture haptics (n = 9; 5.17%), IOL removal (n = 5; 2.87%), anterior vitrectomy with IOL removal (n = 3; 1.72%), nucleus drop (n = 10; 5.7%), IOL explanation (n = 3; 1.72%), IOL explanation with SFIOL (n = 3; 1.72%), lensectomy with SFIOL (n = 8; 4.5%), rescued IOL (5; 2.87%), Pupilloplasty with SFIOL (n = 5; 2.87%), IOL explant (n = 1; 0.57%), Iridodialysis (n = 2; 1.14%), Intravitreal-kenacort (2; 1.14%), PCIOL removal (n = 1; 0.57%), IOL exchange (n = 1; 0.57%), Refixation (n = 2; 1.14%), Lensectomy with pupilloplasty (n = 3; 1.72%), and Lensectomy with PPV and SFIOL (n = 1; 0.57%).

**Table 5:** Details of surgical procedures.

Techniques applied	Values (n %)
PPV + SFIOL (FLAP)	100 (57.47%)
PPV + SFIOL (FLAPLESS)	10 (5.74%)
**ADDITIONAL PROCEDURES**	
PPV + IOLEXPLANATION + SFIOL (FLAP) + SUTURE HAPTICS	9 (5.17%)
PPV + IOL REMOVAL + SFIOL	10 (5.74%)
ANT VITRECTOMY + IOL REMOVAL + SFIOL	3 (1.72%)
NUCLEUS DROP + PPV + SFIOL	10 (5.7%)
PPV + IOL EXPLANATION + SFIOL	3 (1.72%)
IOL EXPLANATION + SFIOL	3 (1.72%)
RESCUED IOL	5 (2.87%)
LENSECTOMY + SFIOL	8 (4.5%)
PUPILLOPLASTY + SFIOL	5 (2.87%)
IOL EXPLANT + PPV + SFIOL	1 (0.57%)
PPV + SFIOL + IRIDODIALYSIS	2 (1.14%)
PPV + SFIOL (FLAP) + INTRAVITREAL KENACORT	2 (1.14%)
PCIOL REMOVAL + SFIOL (FLAPLESS)	1 (0.57%)
IOL EXCHANGE + SFIOL	1 (0.57%)
REFIXATION OF SFIOL	2 (1.14%)
LENSECTOMY + PUPILLOPLASTY + SFIOL	3 (1.72%)
LENSECTOMY + PPV + SFIOL (FLAPLESS)	1 (0.57%)
TOTAL	N = 174 (100%)

**N.B.:** PPV: Pars plana vitrectomy, SFIOL: Scleral fixation of an intraocular lens, IOL: Intraocular lens, ANT: anterior.

## 4 Discussion

Poor capsular support can be observed in a variety of diverse eye disorders, including trauma and pseudoexfoliation syndrome, as well as ophthalmological diagnostic criteria disorders, including Marfan and Weill-Marchesani syndrome. Over the years, many surgical procedures have been established to overcome IOL integrity owing to the apparent lack of an intact capsule. To address such IOL support, ophthalmic surgeons could choose ACIOL, iris-fixated IOL, and SFIOL. However, the percentage of complications and visual acuity recovery differed among the various lines of study. Although earlier findings supported and assessed the efficiency of secondary IOLs, it was remarked that even more findings are needed to establish the superiority of one lens type and fixation site over another. Each of the existing techniques has benefits and disadvantages that must be defined to highlight visual success and complications. Although initiatives for vitreoretinal surgery progress and more records about PPV indications have been revealed, the proportion of cataract extraction following PPV is expanding. However, our findings presented intraoperative, early, and postoperative surgical complications, as well as BCVA outcomes from IOL scleral fixation with PPV. Even though Yamane's approach was specifically described as a "secondary surgery" for the aphakic subject's recovery, our consolidated PPV and SFIOL strategy incorporates numerous ophthalmic concerns in a single sitting, such as a subluxated and dislocated cataract lens involving lensectomy and vitrectomy, a dislocated IOL requiring explanation, PPV and SFIOL implantation within the same sitting. Therefore, it eliminates the necessity for potential surgical complications and operating costs associated with a secondary procedure. Further, as a difference from Yamane's approach, we have not followed the conventional sutured scleral fixation technique, and three-piece IOL was used for SFIOL adopting the Agarwal technique [35]. Optical treatment of monocular aphakia subjects is a therapy challenging task and restoration of visual function in an aphakic patient is difficult. Our research showed these combined surgical approaches were a successful choice for secondary IOL implantation in aphakic scenarios, producing better visual and refractive benefits with manageable postoperative complications.

In contrast to many other methods involving ACIOLs and iris-fixed IOLs, scleral fixation of PCIOLs could indeed preclude a number of postoperative complications because the IOLs are aligned in a more physiologic location and therefore pose a relatively low chance of developing intimate contact with the iris and a considerable distance from the corneal endothelium. In clinical practice, SFIOL is currently the most useful approach since it is stable. This selectivity may be accounted for by the fact that even a vitreoretinal, as opposed to an anterior chamber, feels more at ease functioning behind the iris plane and around the pars plana. Prior to fixing the IOL at the sclera in each of our studies, PPV has been carried out. This enhanced function by discarding all debris, blood, and inflammatory cells from the vitreous cavity, which are either frequent in post-traumatic or complicated post-cataract procedure set up [36].

A single-stage surgery could reduce anesthesia-based comorbidities, surgically induced astigmatism based on scarring, postoperative vulnerability to endophthalmitis, fluctuations in IOP, and the potential signs of retinal breakage and detachment. Apart from managing combined complications related to the vitreoretinal interface, comprehensive vitrectomy assists in avoiding residual vitreous entanglement around the SFIOL, which tends to exacerbate IOL kinking. Extensive vitrectomy can sometimes inhibit the turbulence and traction that cause macular edema and retinal tear. Numerous issues attributed to the residual vitreous, such as epiretinal membrane formation and CME, can be managed to avoid exfoliation of the vitreous and extraction of each capsular component. Furthermore, both complete vitrectomy and anterior vitrectomy have demonstrated comparable success with regard to visual restoration and have benefits and drawbacks [37]. However, the final BCVA was significantly enhanced in our study, and the refractive output was acceptable in all case scenarios.

In our investigation, out of 174 subjects, 110 (63.2%) attained post-operative BCVA of 0.3 LogMAR or better. This is relatively comparable to Lee and Yuen's findings [38], which revealed that out of 25 cases, 19 (76%) had a BCVA of 0.3 logMAR or better. Ghanem and collaborators [39,40] showed that 10 (71.43%) out of 14 eyes that underwent scleral fixation of IOL had postoperative BCVA of 0.2 logMAR or better. Parallel to this, Ozdek and coworkers [41] reported that 14 eyes (93.3%) under scleral fixation of IOL showed significant improvement of postoperative BCVA of 0.3 logMAR or better. The distinction in our sample is that participants with concomitant lesions, such as RD, and the ruptured globe, were not precluded from our research. In these instances, other extensive ocular lesions, contrary to the IOL implantation procedure, have the biggest impact on the final functional prognosis. The primary benefit of surgery was an enhancement in BCVA in each individual in our analysis.Numerous study reports carefully consider multiple strategies for secondary IOL placement, such as iris-fixated, sutureless scleral-fixated, and ACIOL methods, and glanced reported rates of IOP ranging from 12.4% to 4%, IOL dislocation range of 0–12%, hyphema in range of 4.0–9.7%, vitreous hemorrhage range of 0–12.2%, choroidal detachment range of 1.3–2.7%, IOL capture within uveal tissue range of 0–8.6%, CME range of 0–6.9%, RD range of 0–2%, and uveitis in range of 1.1–5.4% [[42], [43], [44], [45], [46]]. Our study also indicates the same ranges of postoperative complications, but the number of eyes and the follow-up duration taken into consideration for observation were not the same in all previous similar studies. However, the strength of our findings is interesting due to the longer follow-up time with more available subjects, and many new postoperative complications with variable frequency, which were lacking in the previous reports. The existing new surgical complications with variable frequency rates were observed, including sphincter tear (n = 6; 3.44%), primary glaucoma (n = 1; 0.57%), secondary glaucoma (n = 13; 7.47%), zonular dehiscence (n = 3; 1.72%), RPE changes (n = 3; 1.72%), iris coloboma (n = 2; 1.14%), choroidal coloboma (n = 2; 1.14%), posterior synechia (n = 2; 1.14%), corneal decompensation (n = 1; 0.57%), macular hole (n = 1; 0.57%), mild NPDR (n = 3; 1.72%), and mild NPDR with diabetic macular edema (n = 1; 0.575). Furthermore, our findings confirmed that the most frequently observed common complication was corneal edema (9.19%), followed by secondary glaucoma (7.47%). In terms of IOL stability, two cases of posterior dislocation were observed during the follow-up period. However, in contrast to earlier studies, we noticed that there were no indications of IOL capture within uveal tissue following SFIOL implantation. Further, the suture needle was inserted through the sclera more vertically perpendicular to the sclera, directed to the posterior pole, and oriented slightly more posteriorly to decrease the chances of optic-iris capture. Our observation represents one case of a macular hole that was not addressed in many previous studies and strongly suggests that it must be considered during the surgical approach in future studies. The early and late surgical complications in our study were comparable to those reported in earlier studies. However, some new complications were observed (Table 6). Additionally, our technique aimed to enhance visual outcomes, decrease comorbidities, stabilize IOL fixation, lessen the damage to the ocular surface, and reduce the surgical time; this approach eliminates the requirements of conjunctival and scleral-based dissection, sclerotomy, suturing of wound closure, and even knotting. In contrast to what was observed in a prior investigation [50], our postoperative results showed a satisfactory refractive outcome that was considerably closer to the targeted refraction.

**Table 6:** Comparison of surgical complication rates among various studies.

Complications	*Kelkar* et al. [25] (n = 31) n%	*Scharioth* et al. [47] (n = 63) n%	*Kumar* et al. [48] (n = 53) n%	*Yamane* et al. [2] (n = 100) n%	*Shelke* et al. [49] (n = 47) n%	Our study (n = 174) n%
Corneal edema	None	5 (7.94%)	–	1 (1%)	2 (4%)	16 (9.19%)
Vitreous hemorrhage	1 (3.2%)	2 (3.17%)	None	5 (5%)	2 (4%)	3 (1.72%)
Hypotony	None	1 (1.59%)	–	2 (2%)	1 (2%)	1 (0.57%)
High IOP	12 (39%)	2 (3.17%)	None	2 (2%)	2 (4%)	8 (4.59%)
Retinal detachment	–	–	–	None	1 (2%)	1 (0.57%)
Cystoid macular edema	None	1 (1.59%)	4 (7.5%)	1 (1%)	2 (4%)	1 (0.57%)
Zonular dehiscence	None	–	–	–	–	3 (1.72%)
Macular hole	None	–	–	None	None	1 (0.57%)
Iris capture	None	1 (1.59%)	–	8 (8%)	1 (2%)	None
Hyphema	–	–	2 (3.7%)	–	–	2 (1.14%)

N.B.: IOP: Intra ocular pressure.

Although our subjects seemed to have a complex range of pre-existing ocular surgical indications, the difference in preoperative to postoperative BCVA was statistically significant (p < 0.0001). In addition, there was no severe sign of increased SFIOL decentration and the case of severe surgical complications after a long-term i.e. 14.2 months follow-up duration in our study period, and the same results were obtained when we applied this technique to non-traumatic aphakic subject eyes due to lack of proper capsule support. IOL dislocation followed by suture breakdown is a significant event that leads to an abrupt visual impairment. The possibility of this problem is still present because our subjects' most recent control visit was 6 months, and most investigations only report it between 2 and 5 years after surgery [51]. Moreover, the rate of knot-related complications varies between studies, and our findings strongly suggest that even existing scleral flaps do not entirely prevent knot erosion over the long term. As a result, a combination of PPV with SFIOL is an effective process to manage IOL and lens dislocation, and even aphakia entirely as a single surgical intervention. Furthermore, our study also highlighted the fact that there were no serious long-term effects and postoperatively short-term choroidal detachment was observed without any detrimental effects on the final visual restoration. Subsequently, prospective future studies comparing the surgical complication rates along with visual acuity success and duration of surgery, employing the pre-conventional techniques against the recently modified techniques are significant to verify and clarify the advantages of the recently used modified surgical techniques. Our investigation revealed diagnostic findings in employing SFIOL in combination with PPV, which emerged to be both safe and effective for improving postoperatively visual outcomes in which capsular support is entirely inadequate and lacking.

## 5 Conclusion

Overall, our findings suggest that these combined surgical approach offers positive results, without increasing the risk of visual acuity and major comorbidities. With significantly higher rates of cataract progression following PPV, subjects with coexisting visually significant cataracts might benefit from combination surgery to overcome visual acuity issues. It is a viable technique for aphakia therapy in subjects with ocular cataracts following primary PPV. Aphakic eyes may benefit from our minimally combined approaches. However, in the case of PPV, a recent focus on new IOLs placement procedures must be adopted to reduce long-term issues without a negative impact on visual gain.

## Author contribution statement

Conceived and designed the experiments: G Nageswar Raoa; Sonu Kumar; Arttatrana Pal.

Performed the experiments: G Nageswar Raoa; Sonu Kumar; Nidhi Sinha; Bhumika Rath; Ar …

## Data availability statement

Data will be made available on request.

## Funding

This research received no specific grants from any funding agency in the public, commercial, or not-for-profit sectors.

## Declaration of competing interest

The authors declare that they have no known competing financial interests or personal relationships that could have appeared to influence the work reported in this paper.
